# Addressing the Confusion within Periodization Research

**DOI:** 10.3390/jfmk5030068

**Published:** 2020-08-28

**Authors:** W. Guy Hornsby, Andrew C. Fry, G. Gregory Haff, Michael H. Stone

**Affiliations:** 1 College of Physical Activity and Sport Sciences, West Virginia University, Morgantown, WV 26505, USA; 2 Osness Human Performance Laboratory, University of Kansas, Lawrence, KS 66045, USA; acfry@ku.edu; 3 School of Medical and Health Sciences, Edith Cowan University, Joondalup 6027, Australia; g.haff@ecu.edu.au; 4 Center of Excellence for Sport Science and Coach Education, SERK, East Tennessee State University, Johnson City, TN 37614, USA; stonem@etsu.edu

**Keywords:** periodization, training, sport science

## Abstract

In this editorial, we focus on recent problematic developments in sport science, and more specifically, problems related to periodization research. Primary areas discussed are (1) appreciation of history, (2) considerations for training studies, (3) the development of concepts, and (4) programming-driven training models.

## 1. Introduction

Over the last several years, disturbing trends related to sport science research and education related to the periodization of training have been noted by the authors of this editorial. Specifically, it appears that there is an increased push by researchers to promote “outside the box thinking”. Certainly, questioning poorly supported dogma and popular belief(s) is at the root of the scientific process. However, for areas of research that have a solid underpinning supported by good scientific data and considerable previous work, appropriate appreciation and understanding should take place before a dismissal of these works occurs. This does not mean that previous findings cannot be challenged or cannot be further developed, as this is a key aspect of the scientific process. However, we believe that most accepted conceptual paradigms of basic and applied science, including sport science, are firmly rooted in logical reasoning and supporting evidence. We also believe that most good conceptual paradigms develop and evolve over time as a result of critical thinking, sound research (often painstaking), and a diligent search for clarity. As such, a central goal of this editorial is to inspire future research. 

A troubling current trend in academics appears to be an “everything we have been taught is wrong” attitude, carried out with an intent to create controversy, and this trend has spilled over into sport science research, particularly as it pertains to periodization. Perhaps some (much?) of these issues are related to/influenced by social media (of which we readily admit that we are not experts). Researchers are now more connected virtually, with others across the globe leading to constant commentary, for better and for worse. Several of the authors have been scientists for a long time (>30 years), and we unanimously feel that today’s students and young scientists are developing in a climate that is too focused on self-promotion and too often misses the big picture. Over the last several years, we have read many review and opinion papers [1,2,3,4] on periodization in which we feel the questioning is far too simplistic (e.g., questioning a referenced statement or using a circumscribed thought process more common in the basic sciences) and misses the breadth and robustness of the historical development of periodization (which is well beyond the scope of this editorial). Thus, we feel it is worth briefly discussing the current climate of periodization research/commentary and addressing several aspects of several specific issues. Below are three primary issues related to periodization research followed by a brief commentary on a newer development within the field. 

## 2. Appreciation of History 

Recently, it seems that a more commonly held belief has arisen that “older” research is out of date and less helpful than recently published research. This can result in authors selectively choosing references or statements; potentially missing important work; or worse, specifically choosing studies that fit one’s personal narrative. It is particularly surprising how research in the areas of resistance exercise, strength-power development, etc., performed in 1970s, 1980s, and 1990s was perhaps the biggest “lift” in knowledge the field has experienced [5], and yet many authors only cite work performed in the last 15 years. As authors we have, on several occasions, been told by reviewers to replace a reference of an older study with a more current study or even provided the suggestion that a reference is “too old”, apparently dismissing older work. It is worth noting that if only current research is valuable, that means that what we are doing today will eventually become obsolete and valueless. We believe an appreciation of historical work may be helpful for limiting the cyclical, “re-invention of the wheel” nature of some training and research practices. 

A lack of appreciation of older literature may lead to improper citing of that older literature (when it is cited) or at the very least lead to misinterpretation of this research. Perhaps most importantly, there does not seem to be an appreciation for the development and evolution of ideas and concepts. We have been particularly dismayed at several recent papers that have used details from older references to dismiss entire concepts that evolved from these initial studies [1,2]. Additionally, we often read a basic statement regarding an aspect of periodization suggesting that it is relatively simplistic and straightforward information when in fact it is much more layered and complex [1,2,3,4]. 

One example of a common oversimplification is the reference of Lenoid P. Matveyev (USSR) being recognized as the “Father” of periodization. While Matveyev did formalize the periodization conceptual paradigm, he clearly built on previous work and contributed to the evolution of the paradigm. The historical development of periodization has a long and rich history dating back several centuries to the ancient writings of Philostratus; Galen; Avorroes; and, more recently, L. Pihkala (1930s–1950s, Finland), L. Nadori (1940–1960s, Hungary), and N.G. Ozolin (1940s–1960s, Soviet Union). A few of the earliest texts, written in English, that describe breaking up the training plan into “periods” include books by G. Dyson (A New System of Training, 1946), W. Bresnahan and G. Tuttle (Track and Field Athletics, 1947), and K. Doherty (Modern Track and Field, 1963) [6,7]. While these are only a few examples within the vast history that underpins the theory of periodization, it demonstrates that prior to Matveyev’s seminal contribution to the topic there was in fact foundational work being completed [6]. For an incredibly in-depth look into the history of periodization (particularly for track and field.), we refer the reader to Bourne, 2008 [6]. 

Indeed, Matveyev’s contribution to periodization, particularly his observational work on periodization monitoring of the Soviet track and field athletes preparing for the 1952 and 1956 Olympic Games, and later, his text describing an annual training plan (1965), cannot be underappreciated [8]. It is worth mentioning that Matveyev was observing athletes being coached by full-time coaches who were creating the training plans (not Matveyev), and likely the reason for the popularity and attachment (credit) to the traditional (or classic) model of periodization is due to the 1965 text, Periodization of Sports Training, being translated into English. For this textbook and the periodization paradigm, many of the underlying mechanistic concepts had been previously developed by H. Selye, N.N.Yakovlev, and I.P. Pavlov [9]. 

## 3. Considerations for Training Studies 

Discussions on the importance of the applied nature of sport and thus aspects related to that understanding are well documented [10,11]. Too often in studies in which interventions are compared, the interventions do not occur in a manner reflecting real-world application. We appreciate the “tug of war” that sport scientists often face, trying to juggle internal validity (in an effort to control certain aspects) with real-world parameters. However, common efforts to control certain aspects of a study, such as equating volume when comparing two different training strategies (likely changing 1 or both programs from what typically occurs), or using a smith machine to control inter-subject technique differences, etc., should be well addressed in the limitations and practical application portion of a manuscript. 

When discussing short-term training studies (the duration of most training interventions) [12], it is important to consider that 2–3 months is only a blip in an athletes training life. Thus, periodization pioneer Yuri Verkhoshansky explains, in the highly influential sport training textbook “Supertraining”…

*“Virtually any method of strength training will enhance the strength of a novice during the first few months, provided the intensity, in particular, is kept at a safe level. This is a major reason why it is misleading and counterproductive to apply the results obtained from scientific studies of less than six months’ duration. It is also a major reason why relatively inexperienced coaches manage to have initial success with athletes…” [6].*

We certainly appreciate how difficult long-term periodization studies are, such as [13,14,15], as most of our work, with a few exceptions [16,17], is rooted in semester length designs. We feel too often short term (6–10 week) training studies are simply viewed as “what worked better at developing performance adaption(s)?” (often, carried out on untrained or minimally trained subjects) vs. a more in-depth context such as efficiency of training [17]; the amount of work (and thus, fatigue) necessary to maximize results; and timing and direction of training, a coaches ability to direct and control the training process at certain time points [18,19]. We continue to be surprised by the apparent disregard for highly ecologically valid athlete monitoring studies capturing trained athletes in real-world environments [16,19,20,21] (these are only a few in a long line of research). We fully understand the limitations of causation (no comparison group, a “non-normal” sample); however, we believe this is some of the most helpful information for coaches and sport scientists related to periodization as well as other aspects of training. 

Another important consideration is the amount of detail related to the training that is performed. Mujika [22] has previously called for authors of training studies to report volume load and detailed information of the training prescription. This is critical, as many times based upon the training information provided it is virtually impossible to replicate the intervention conducted. We believe that journals should request that authors report how the training was verified (e.g., were the investigators observing? Were they strength coaches? Was it a sport Coaches report? A self-report?). It is perhaps not surprising that direct observation is superior to reporting from subjects [23]. Additionally, it would be helpful for authors to state whether or not the training reported was the planned prescription, beforehand, or if the training was recorded as it was carried out. 

Lastly, most training studies referred to as periodization studies are really programming studies (see discussion below), as the manipulation and comparisons of different strategies are usually dictated by differences in set-rep schemes, intensities, etc., and not by over-arching timelines or adaptation-based fitness phases and goals. 

## 4. The Development of Concepts 

Training concepts (e.g., training theory) can be astonishingly multifaceted and complex. Sometimes in attempting to summarize basic, translational and applied science, the large amount of physiology that goes into training concepts (e.g., fitness–fatigue paradigm, General Adaptation Syndrome, etc.) is lost. Related to bullet point #1 (history), to truly appreciate an established training concept one must really know the historical literature. Additionally, for sport research it is also likely helpful to understand aspects well beyond the literature (coaching, training, sport, and history). For example, using a very specific issue, that is a relative component within a larger concept, and then dismissing the entire concept as a mistake: disregarding a mechanistic concept such as Selye’s work on stress response because his original studies were not based on exercise as a paradigm and therefore suggesting that the General Adaptation Syndrome (G.A.S.) cannot be used conceptually in explaining the response to exercise and training [24]. Training concepts can be quite helpful for sport scientists and coaches, as they allow complicated information from multiple scientific disciplines to be synthesized for useful application [24,25]. Perhaps a worthwhile analogy is the comparison of athlete skill to technical models of performance for various sport tasks (e.g., stages of sprinting or of the snatch exercise). The summarizing nature of a technical model (or a training concept) can almost assuredly be questioned and picked apart to some degree. For example, it could be pointed out that someone incredibly successful (e.g., a gold medalist) did not perform in the exact manner recommended by the technical model. Interestingly, it is quite commonly accepted that technical models have nuance and “ranges”. This same degree of acceptance does not seem to hold for periodization [3,4]. 

## 5. Programming Driven Training Strategies

Lastly, we have read several articles and frequently observe discussions on social media incorrectly portraying the idea that block periodization (or traditional periodization for that matter) is stagnate and that the programming within the periodized plan cannot be modified if needed during its implementation. Recently, there has been an increased push to use a training strategy that is more in-the-moment-focused. Many terms have been used to describe these programming centered training plans that involve a day to day organization template, for example, agile periodization, flexible periodization, and fluid periodization [26,27,28]. Instead of a long-term, detailed training prescription, this approach to training involves a format in which training is dictated by an athlete’s alleged current state (e.g., readiness, and fatigue, which are often subjective), and to a large degree, based on a certain selection format, assembled session to session. Conceptually, these training models are not periodization models and are actually programming models, thus including the term periodization is a misnomer [24]. This is because these flexible training models are driven by day-to-day and week-to-week programming decisions, often based on athletes’ subjective feelings and not objective evidence or an over-arching periodization strategy. Periodization is a conceptual outline dealing with timelines and fitness phases; depending upon the goal of the training process, it creates time-direction of training volume, intensity, and task specificity factors [25]. Programming drives the periodization phases (makes the phases within the periodized plan happen) and includes exercise selections, loading parameters, rest periods, etc. [24,25]. However, using a periodization model allows substantial programming modifications to be made [25]. It should be noted that programming alterations should be made based upon valid and reliable evidence concerning the state of the athlete, evidence that can be provided by a well-designed athlete monitoring process [29]. Indeed, programming alterations should be based on good data; a well-conceived, integrated long-term plan; and in most cases, be subtle. 

Within U.S. Collegiate sport and Australian professional sports, we continue to be surprised by how often we hear “periodization doesn’t work for team sports” or the more general statement “periodization doesn’t work for my situation.” We have speculated that perhaps avoiding this long-term planning approach is due to how strength coaches are often placed in a servant type role to the head sport coach [30], and perhaps the idea is that these more flexible approaches allow the strength coach to work around the head sport coach. We believe this thinking is flawed, in that (1) attempting to manage a more unpredictable situation with more unpredictability is a mistake, and (2) the programming within a periodized plan can be altered if need be, while being guided by the overall goal(s) of the given training phase. Or, as more eloquently stated by Bourne [6], “the use of periodization is synonymous with a scientific approach to training as coaches, scientists, and athletes attempt to gain maximal control over the variables affecting the adaptive process.”

## 6. Conclusions 

Recently, we (along with many colleagues) authored a letter to the editor (LTE) [31] addressing what we felt were issues in a recent article by Buckner et al. [2]. In the same issue of Medicine and Science in Sports and Exercise, Buckner et al. responded to our LTE [32]. For a much better picture of the overall discourse, we refer the reader to those papers, which address several important concepts of strength development [2,31,32]. Of particular note are some of the statements made in the follow up by Buckner et al. [32] regarding periodization that we thought were worth addressing. We appreciate that skeptics are acknowledging the complexities of periodization, and this is an important step in understanding and interpreting the scientific literature. However, simple blanket statements seldom explain the intricacies of advanced training methods for high-level performance. Although there is certainly much work remaining, characterizing the arguments as “everything is periodization”, or that “periodization has not been well studied” or “appropriately studied”, suggests an unfortunate lack of understanding of the topic. Additionally, an appreciation of the differences in training advanced and elite-level competitors with long training histories compared to the very forgiving population of untrained or novice individuals, who readily respond to almost any training stimulus, is essential for making sense of the available data. The ability to implement scientifically-based training programs with populations of all training and performance levels, and to work closely in concert with the actual coaches and trainers, is certainly a critical part of the process. Unlike the researcher who is often simply searching for statistically significant results, or evidence of a recognizable training effect, the effective sport scientist, coach, and trainer strives for optimum performance results at competition time. Recognizing and developing all facets of successful performance is critical to achieve the desired consistent and long-term results, whether it is on the athletic field or in any occupation requiring high levels of performance. Only through the application of these training paradigms with actual high-level performers can the external/ecological validity of the training methods be properly established. This discourse on the topic of periodization certainly validates our long history of careful study of this complex topic, and we eagerly look forward to conducting many future studies related to periodization. Like with many research topics, those who produce the scientific data on the efficacy of periodization quickly appreciate the inherent challenges of this line of inquiry. In 1910, former U.S. President Theodore Roosevelt may have summed it up best in one of his most famous speeches given at the Sorbonne in Paris, France, where he cautioned of individuals who have “a readiness to criticize work which the critic himself never tries to perform” [33]. It is hoped that authors eager to produce numerous reviews and commentaries on this and related topics can supplement their work with empirical research data from their labs, and we invite them to be part of the constructive effort to properly understand the science of periodization and programming.
